# A Miniaturized Multiband Antenna Array for Robust Navigation in Aerial Applications

**DOI:** 10.3390/s19102258

**Published:** 2019-05-16

**Authors:** Stefano Caizzone, Georg Buchner, Mihaela-Simona Circiu, Manuel Cuntz, Wahid Elmarissi, Emilio Pérez Marcos

**Affiliations:** Institute of Communications and Navigation, German Aerospace Center (DLR), 82234 Wessling, Germany; georg.buchner@dlr.de (G.B.); mihaela-simona.circiu@dlr.de (M.-S.C.); manuel.cuntz@dlr.de (M.C.); wahid.elmarissi@dlr.de (W.E.); emilio.perezmarcos@dlr.de (E.P.M.)

**Keywords:** GNSS, antenna, antenna array, robustness, interference, UAV, jamming

## Abstract

Satellite navigation is more and more important in a plethora of very different application fields, ranging from bank transactions to shipping, from autonomous driving to aerial applications, such as avionics as well as unmanned aerial vehicles (UAVs). Due to the increasing dependency on satellite navigation, the need for robust systems able to counteract unintentional or intentional interferences is growing. When considering interference-robust designs; however, the complexity increases. Top performance is obtained through the use of multi-antenna receivers capable of performing spatial nulling in the direction of the interference signals. In particular, mobile applications (aeronautics, UAVs, automotive) have a substantial interest in robust navigation, but they also have the strongest constraints on the weight and available places for installation, with the use of bigger and heavier systems posing a substantial problem. In order to overcome this limitation, the present work shows a miniaturized five element (4+1) antenna array, which operates at the L1/E1 band (with array capability), as well as at the L5/E5 band (as a single antenna). The proposed antenna array is able to fit into a 3.5-inch footprint, i.e., is compliant with the most widespread footprints for single antennas. Moreover, it is capable of multiband operation and meets the requirements of dual-frequency multi-constellation (DFMC) systems. Thanks to its extreme miniaturization and its compliance with current airborne single antenna footprints, the presented antenna array is suitable for easy integration in future aerial platforms, while enabling robustness and enhancing interference mitigation techniques using multi-antenna processing.

## 1. Introduction

The use of satellite navigation is nowadays very widespread and embraces almost all fields of modern life [1]. Beyond being an incredible booster for location-based services, such ubiquitous use of satellite navigation also poses serious risks, due to the increasing dependency of safety-critical systems with respect to global navigation satellite systems (GNSS) [2]. Threats due to unintentional as well as intentional interferences can cause enormous damage [3], both in terms of costs and lives. Countermeasures are currently being deployed worldwide. Multi-antenna receivers could till now prove the best performance. They are able to place spatial nulls in the direction of the interference signals and therefore can limit their effect on the position solution [4,5].

Such systems are capable of suppressing interferences by orders of magnitude stronger than the navigation signals; their drawback, however, is usually their complexity in terms of size, weight, and power consumption.

Such limitation becomes a crucial point for mobile applications, such as airborne ones.

In order to overcome it, different groups have lately been developing miniaturized antennas and receivers [6,7,8,9]; however, the antenna size was still bigger than commercial single antennas (which usually fit into a footprint of 3.5 inches) and therefore their use in civilian airborne applications to date has been limited.

The present work shows a miniaturized five element (4+1) antenna array, with four antennas operating at the L1/E1 band and a single antenna receiving L5/E5a signals. Such an array is able to fit into a 3.5-inches footprint, and is therefore suitable for use in safety-of-life airborne applications, thanks to both its reduced dimensions and to the dual frequency capability which is needed, for example, for ionospheric corrections.

The concept of the proposed antenna array will be shown first with measurements in anechoic chambers validating the simulation results. GNSS measurements performed with the antenna will also show its usability in the satellite navigation context. Moreover, its capability to enable interference suppression will be verified with the help of simulations with typical algorithms for interference mitigation.

Finally, the installed performance of the antenna on top of a commercial aircraft and an octocopter will be analyzed through precise electromagnetic simulations, showing its capability to perform as good as current single antenna systems when used in reference mode (i.e., in the absence of interferences).

## 2. Antenna Array

The basic requirement for the antenna design is the standard 3.5 inches (~90 mm) footprint with four screws for installation.

**Figure 1:**
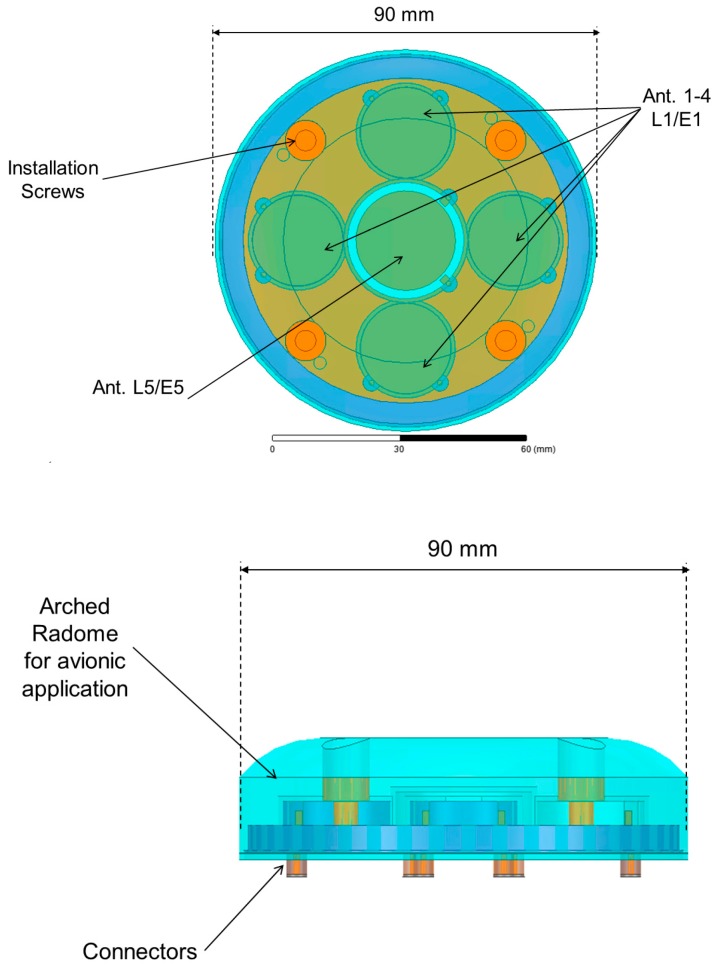
Top view (**top**) and side view (**bottom**) of the antenna array.

Due to the very limited space available, strong miniaturization of the single antennas, as well as an extremely reduced mutual distance between the elements, is required (Figure 1). 

In order to cope with the contrasting requirements of miniaturization and bandwidth, a dielectric resonator antenna technology has been chosen. Each antenna has two feeding pins that excite linear polarizations. Such pins are then connected to broadband hybrid circuits for the generation of Right Hand Circular Polarization (RHCP). The antenna design has been recently patented [10].

The antenna has been simulated using Ansys High Frequency Electromagnetic Field Simulation Software (HFSS). In the simulations, the antenna is surrounded by air.

**Figure 2:**
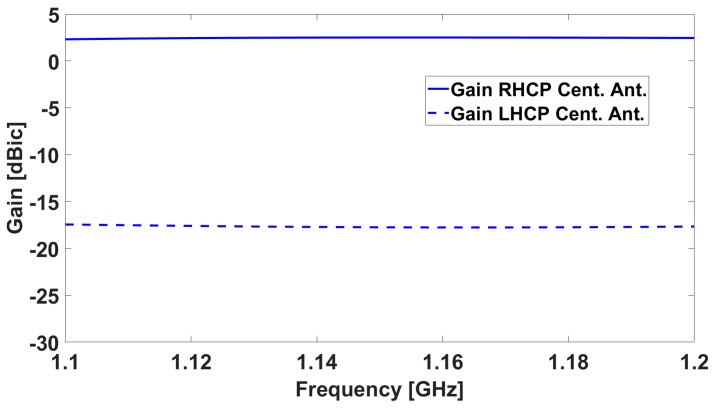
Simulated realized gain for the central antenna element: the solid line is the RHCP, while the dotted line is the Left Hand Circular Polarization (LHCP).

**Figure 3:**
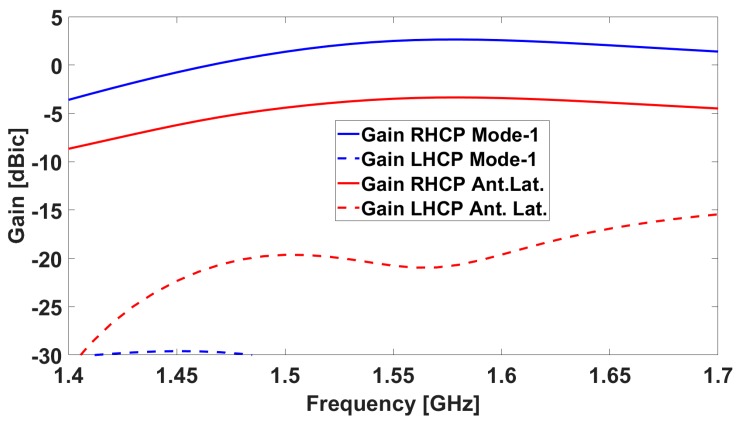
Simulated realized gain for one of the lateral antenna elements (**red**) and Mode 1 (**blue**): the solid line is the RHCP, while the dotted line is the LHCP.

The simulated results for the antenna in terms of realized gain are shown in Figure 2 and Figure 3, both for the central antenna (operating at L5/E5) and for one of the lateral antennas (covering the L1/E1 band). 

Moreover, the antenna can operate in an “array mode” when no interference is detected. In that case, the antenna outputs will be combined constructively in order to obtain a smoother pattern with higher gain levels. We will refer to it as “Mode 1”.

Critical antenna design performance metrics like good matching and low mutual coupling between the antennas (with the maximum mutual S-parameter being ~−12 dBic at the L1/E1 central frequency) are met. RHCP gain at zenith is about 2.5 dBic for the central antenna at the L5/E5a central frequency, while at the L1/E1 band, it is about 2.6 dBic for Mode 1. The lateral antennas, when considered singularly, have an RHCP gain at the boresight of about −3.5 dBic at the L1/E1 central frequency (smaller than the central antenna due to coupling effects, causing pattern distortion with maximum gain not being at the boresight anymore, as shown later) The axial ratio at the boresight of the central frequency is 1.6 dBic for the central antenna and 2.2 dBic for the lateral antennas, respectively.

**Figure 4:**
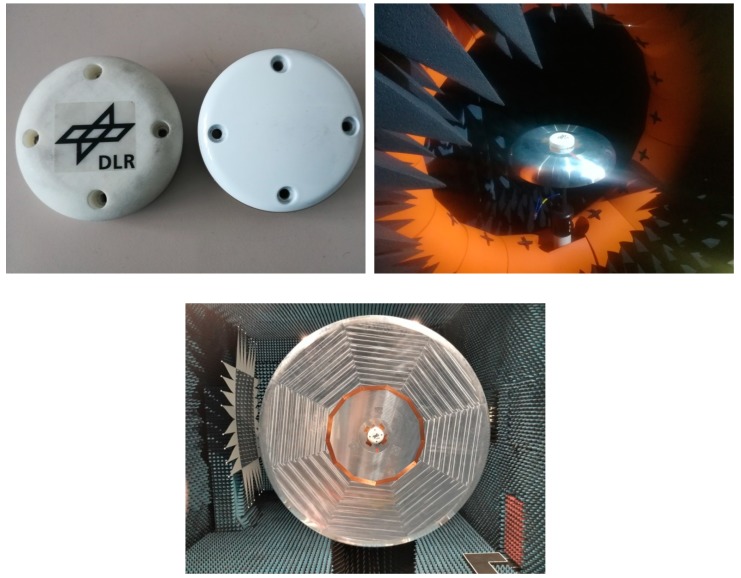
**Top left**: manufactured DLR array (on the **top left**) compared with a commercial avionic single antenna with the same 3.5-inch footprint; **top right**: antenna array placed on the small rolled edges ground plane during the electromagnetic measurement in the semi-anechoic near-field chamber at the DLR facilities; **down**: antenna array, placed on the big rolled edges ground plane, during the electromagnetic measurement in the Compact Test Range at the DLR facilities.

The antenna array has been manufactured (Figure 4) and tested both in a semi-anechoic near field chamber (Satimo Starlab) as well as in the bigger Compact Test Range far-field chamber, both available at DLR. Two different ground planes have been used, having a flat zone with a diameter of 40 cm and 122 cm, respectively; both of them have rolled edges to minimize diffraction effects from the edges of the ground plane. The bigger ground plane was manufactured following the specifications of DO-373 [11]: such a ground plane, however, is heavy and needs anechoic chambers with large quiet zones (as the Compact Test Range (CTR) chamber available on the DLR premises) to be measured. For the sake of comparison, a smaller ground plane (with a 40 cm flat zone diameter) was also manufactured. In this case, the ground plane fit into the smaller anechoic chambers such as the Starlab.

**Figure 5:**
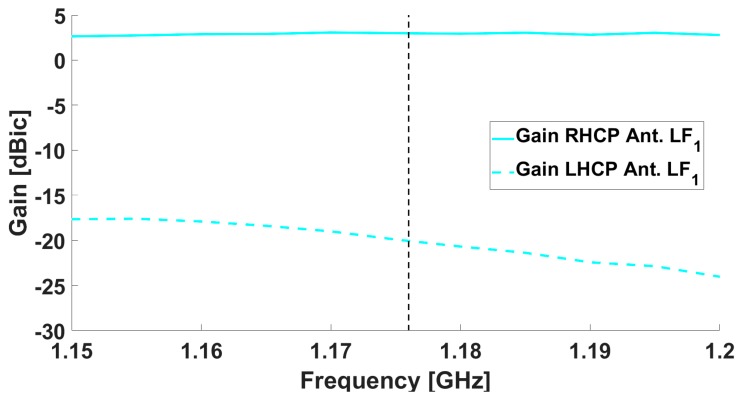
Gain as measured in the Starlab near-field semi-anechoic chamber for the central antenna: the solid line is the RHCP, while the dotted line is the LHCP.

**Figure 6:**
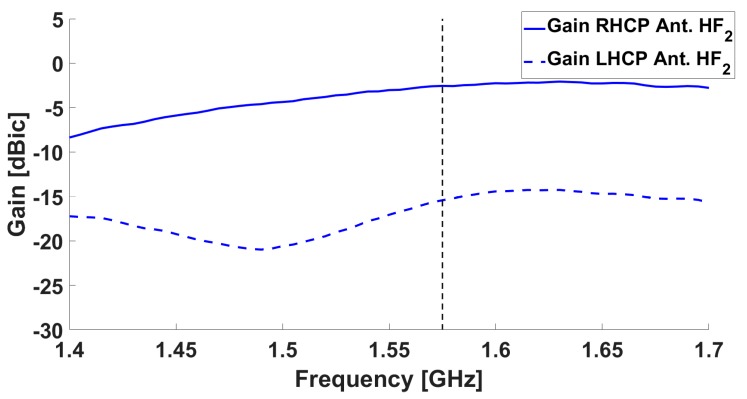
Gain as measured in the Starlab near-field semi-anechoic chamber for one of the lateral antennas: the solid line is the RHCP, while the dotted line is the LHCP.

**Figure 7:**
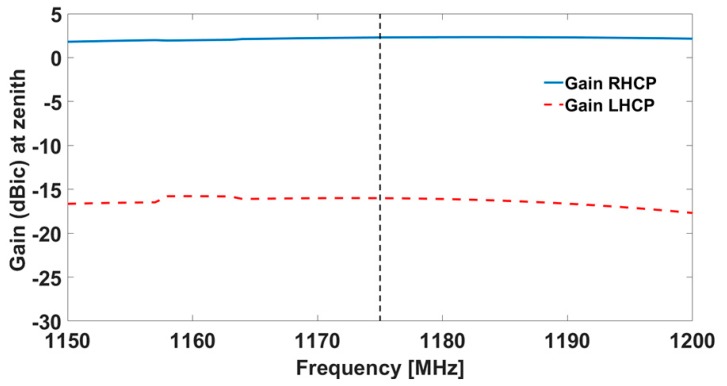
Gain as measured in the CTR chamber for the central antenna: the solid line is the RHCP, while the dotted line is the LHCP.

**Figure 8:**
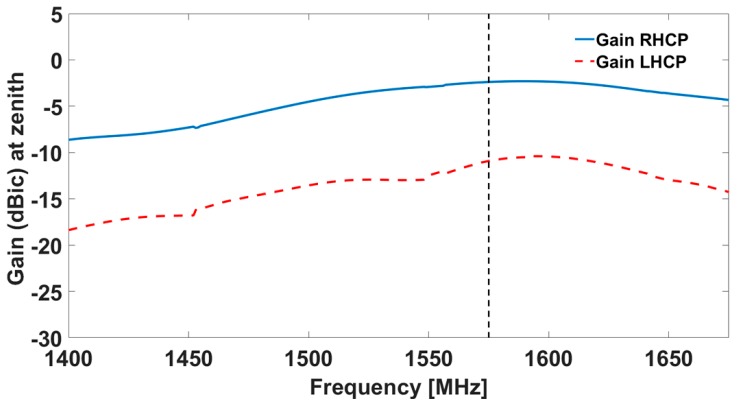
Gain as measured in the CTR chamber for one of the lateral antennas: the solid line is the RHCP, while the dotted line is the LHCP.

Results from the measurements in both chambers / on both ground planes are shown in Figure 5, Figure 6, Figure 7 and Figure 8. The measured results are in good agreement with the simulated ones, shown in Figure 2 and Figure 3. Moreover, the frequency trends appear similar on both ground planes. 

**Figure 9:**
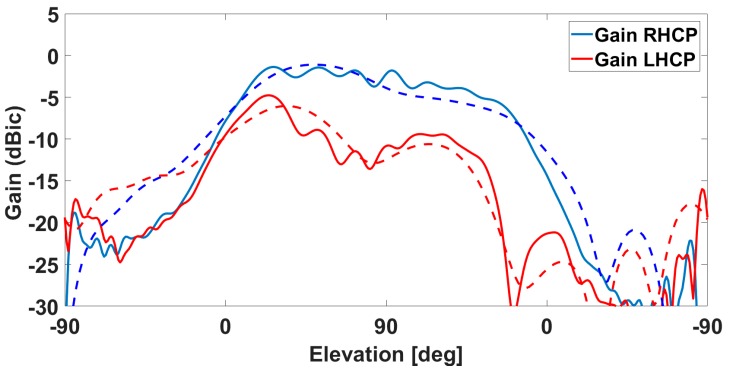
Pattern cut of the gain of one lateral antenna: (**blue**) RHCP; (**red**) LHCP. (**Solid Line**) on big ground plane; (**Dotted Line**) on small ground plane. Ripples in the solid lines are due to reflection from the ground plane itself.

Small differences between the measurements on the two ground planes appear mostly on the LHCP components and when looking at the pattern cuts, as for instance in Figure 9.

They are due to a reflection happening on the surface of the big ground plane. This causes more ripples in the pattern at high elevations (Figure 9), which are however not due to the antenna intrinsic characteristics but only to the overlapping of the reflected waves with the ones originating by the antenna itself (The use of the small ground plane has indeed been suggested recently by the authors to standardize bodies as a more valid approach for the characterization of the antenna in a standalone configuration, i.e., without the specific aeronautic platform on which it will be mounted).

**Figure 10:**
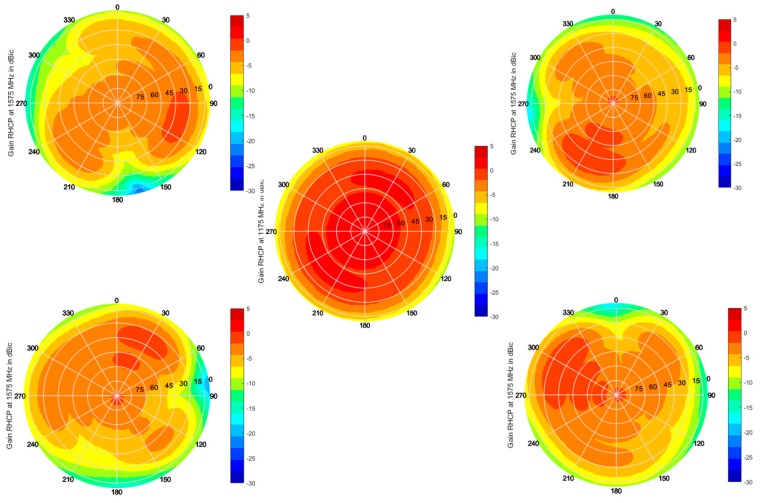
Sky plots of the embedded antenna RHCP gain in dBic, as measured in the anechoic chamber.

**Figure 11:**
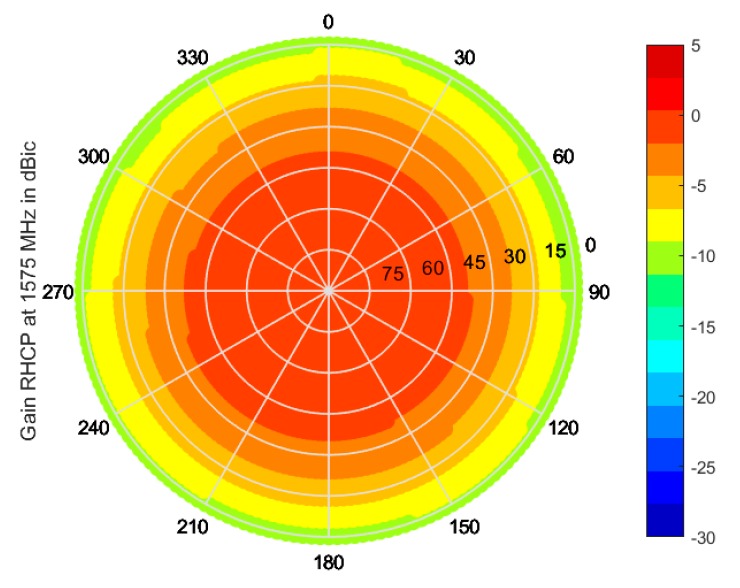
Sky plot of the Mode 1 RHCP gain in dBic, as measured in the anechoic chamber.

Figure 10 shows the sky plots of the single antenna element gain at their central frequencies, i.e., at 1575 MHz for the lateral antennas and at 1175 MHz for the central one. While the center element has a uniform coverage, as expected from a single antenna, the patterns of the lateral antennas are distorted due to the close vicinity of each element with other antennas resonating at the same frequency. Though such an effect does not allow for optimal coverage by each antenna, it ensures that all sky sectors are well covered by at least one antenna. By combining the lateral antenna outputs for Mode 1 operation, moreover, a smooth and uniform coverage throughout the upper hemisphere is achieved, as shown in Figure 11.

## 3. GNSS Measurement of the Standalone Antenna

**Figure 12:**
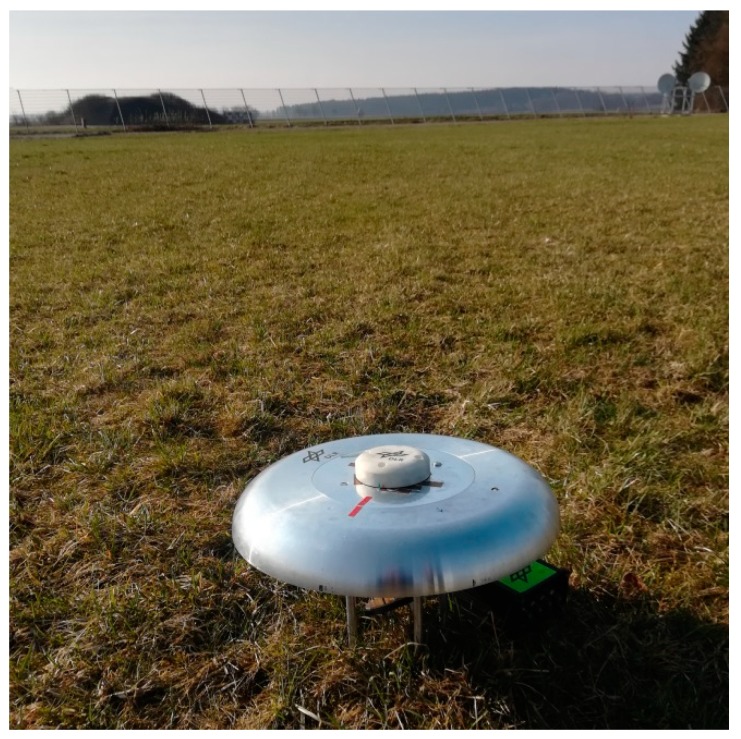
DLR antenna on a small ground plane and connected to a Javad receiver for GNSS field tests.

**Figure 13:**
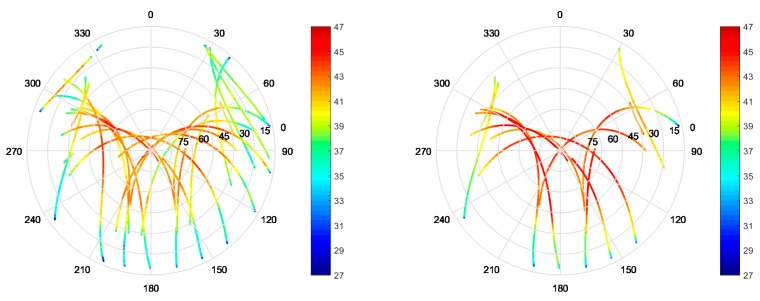
Sky plots of the recorded C/N0 values over the different satellite passes for L1/E1 (**left**) and L5/E5a (**right**).

The proposed antenna has been tested also from a GNSS point of view. For such scope, the antenna alone (i.e., not mounted on any aerial vehicle, but only placed on the small ground plane) was connected to a Javad Delta receiver and placed in an open field on the DLR premises (Figure 12) to minimize multipath effects. GNSS observables and C/N_0_ values on L1 and L5 signals over a time span of 9 h have been collected: the sky plot of the recorded C/N_0_ values can be seen in Figure 13, both for L1/E1 and L5/E5 bands; good reception from the whole sky plot is visible, with C/N0 values of up to 47 dB-Hz at both bands recorded for medium to high elevations.

## 4. Interference Suppression Capability

**Figure 14:**
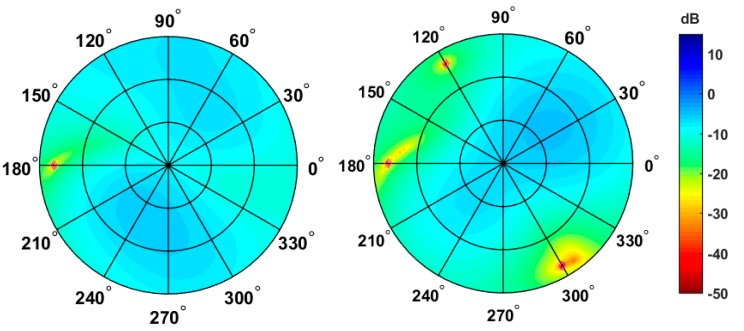
Sky plots of the array gain after applying weights for nulling in the direction of the interferer(s). Interference Direction of Arrival (DOA) marked with a red rhomb. **Left**: one interferer; **right**: three interferers.

In order to show that, despite the extreme miniaturization, the proposed antenna array can properly suppress interferences and hence add robustness with respect to single antenna designs, simulations of the antenna array in conjunction with interference suppression algorithms have been performed. The simulations take into consideration the patterns of each of the antenna elements in the array and several Continuous Wave (CW) interferences. Figure 14 shows the resulting digitally formed antenna-array gain patterns after the interference has been mitigated. On the left side of Figure 8, a single CW interference was simulated, while on the right side, three CW interferences (impinging the antenna-array from different directions) were simulated.

Good interference suppression can be observed for both cases, with the coverage of the remaining sky plot areas becoming, as expected, worse as the number of interferers increases. More details on the use of interference suppression algorithms with miniaturized antenna arrays can be found in [12].

## 5. Installed Performance Analysis

The extreme compactness of the antenna array, as well as its multiband capability, make it particularly suitable for mobile applications, such as airborne ones, where place and weight constraints play a major role.

In order to verify the performance of the antenna array once mounted on different airborne platforms, installed performance simulations have been performed, where the measured antenna characteristics have been integrated with the platform CAD model. In particular, the currents of the antenna array in Mode 1, as measured in the semi-anechoic chamber and reconstructed on a box, have been used as field sources in an electromagnetic simulator, once placed in the installed position on the platform.

**Figure 15:**
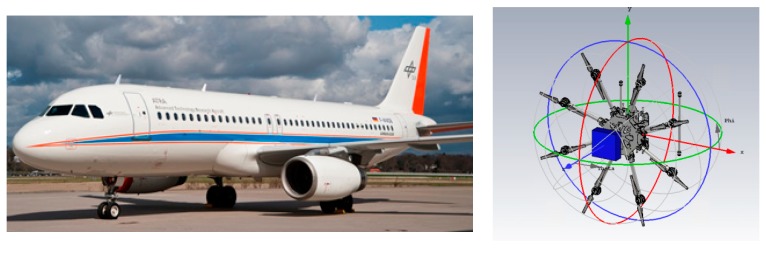
Left: DLR Advanced Technology Research Aircraft (“ATRA”): Airbus A320; right: DJI S1000 drone cad model.

**Figure 16:**
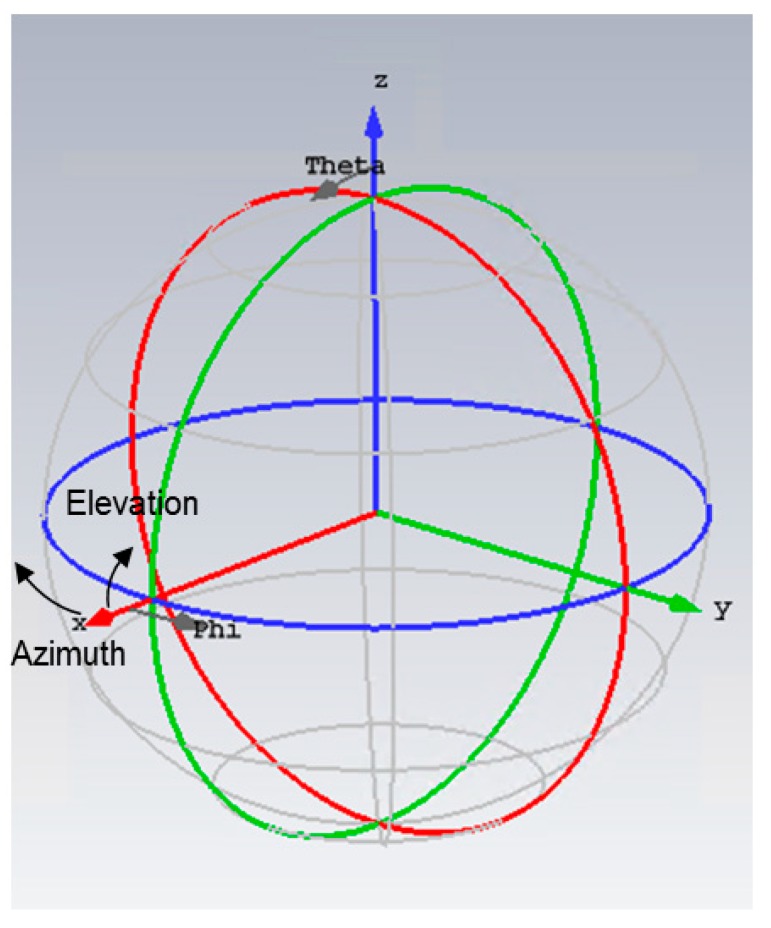
Coordinate Systems for both the GNSS results (elevation and azimuth) and e.m. simulations (phi and theta).

Two examples have been investigated: an aircraft installation and a drone installation. For the aircraft, a simplified model of an Airbus A320 (as the DLR one shown in Figure 15) has been considered. For the drone, a commercial DJI S1000 drone is being considered (Figure 15 right).

(please be aware of the coordinate system being used in this section, which is different from the one commonly used in the GNSS community. The relationship of the coordinate systems (GNSS vs. e.m. simulations) is shown in Figure 16.)

**Figure 17:**
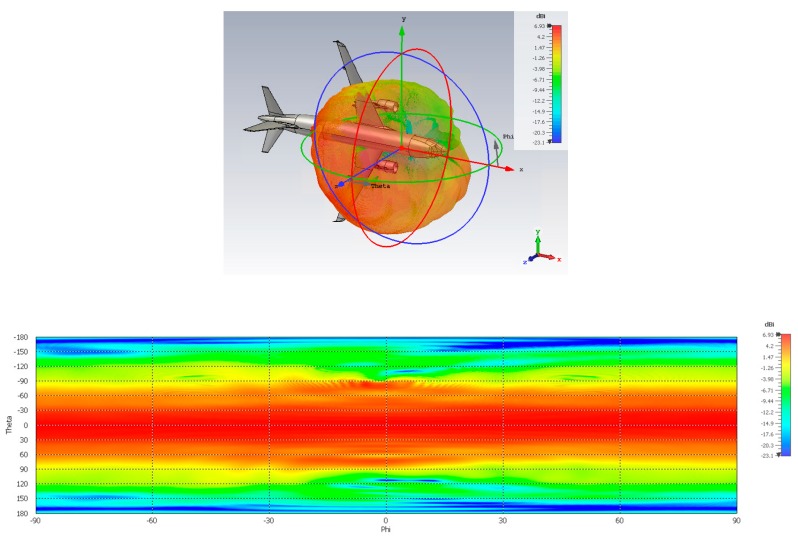
RHCP radiation pattern of the DLR antenna array in Mode 1 once mounted on an aircraft: (**top**) 3D view; (**bottom**) 2D view.

**Figure 18:**
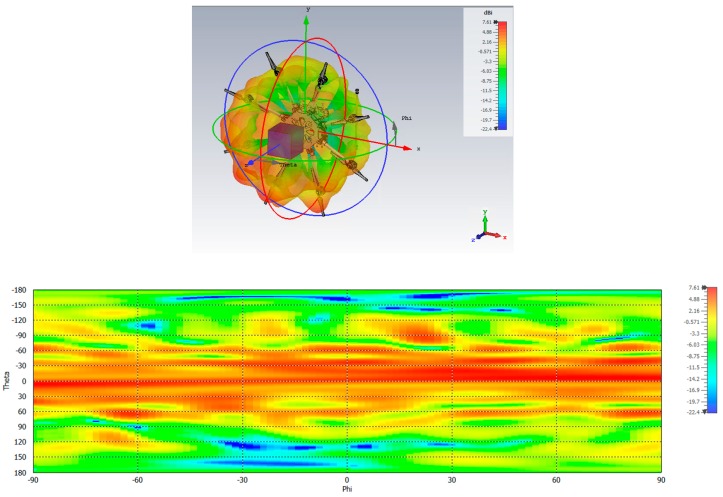
RHCP radiation pattern of the DLR antenna array in Mode 1 once mounted on a drone: (**top**) 3D view; (**bottom**) 2D view.

Good behavior in the case of the aircraft can be observed (Figure 17), with a broad beamwidth enabling optimal coverage of the sky plot in case of no interference. The results for the drone (Figure 18) also show good coverage of the upper hemisphere, with more waviness due to strong scattering from the many metallic parts in the close vicinity of the antenna. Also more backradiation due to the limited ground plane/absence of flush mounting can be observed.

## 6. Conclusions

In this work, a new and miniaturized five-element antenna array (with four L1/E1 elements and one L5/E5 element) fitting into a 3.5-inch footprint has been shown. Both electromagnetic and GNSS tests have validated the antenna suitability for use in mobile applications. It has been demonstrated how, in an interference-free scenario, the antenna array is capable of ensuring good satellite signal reception at the foreseen bands in almost the whole upper hemisphere. Moreover, it has been shown through simulations how the antenna array properly enables interference suppression/mitigation techniques based on digital array processing. Finally, the installed performance of the array once mounted on an aircraft or a drone has been analyzed, confirming its suitability for airborne applications.

## References

[1] Teunissen P.T.J., Montenbruck O. (2017). Handbook of Global Navigation Satellite Systems.

[2] Dovis F. (2015). GNSS Interference, Threats, and Countermeasures.

[3] Günther C. (2013). A survey of spoofing and counter-measures. Navigation.

[4] Cuntz M., Konovaltsev A., Meurer M. (2016). Concepts, Development and Validation of Multi-Antenna GNSS Receivers for Resilient Navigation. Proc. IEEE.

[5] Vagle N., Broumandan A., Lachapelle G. (2016). Analysis of Multi-Antenna GNSS Receiver Performance under Jamming Attacks. Sensors.

[6] Basta N., Dreher A., Caizzone S., Sgammini M., Antreich F., Kappen G., Irteza S., Stephan R., Hein M.A., Richter A. System Concept of a Compact Multi-Antenna GNSS Receiver. Proceedings of the 2012 The 7th German Microwave Conference.

[7] Caizzone S. (2017). Miniaturized E5a/E1 Antenna Array for Robust GNSS Navigation. IEEE Antennas Wirel. Prop. Lett..

[8] JKasemodel J.A., Chen C.C., Gupta I.J., Volakis J.L. (2008). Miniature Continuous Coverage Antenna Array for GNSS Receivers. IEEE Antennas Wirel. Prop. Lett..

[9] Volakis J.L., Brien A.J.O., Chen C.C. (2016). Small and Adaptive Antennas and Arrays for GNSS Applications. Proc. IEEE.

[10] Caizzone S., Elmarissi W., Buchner G., Cuntz M. (2018). Controlled Radiation Pattern Antenne. German Patent Pending.

[11] (2018). DO-373-MOPS for GNSS Airborne Active Antenna Equipment for the L1/E1 and L5/E5a Frequency Bands.

[12] Pérez Marcos E., Caizzone S., Cuntz M., Konovaltsev A., Meurer M. STAP Performance and Antenna Miniaturization in Multi Antenna GNSS Receivers. Proceedings of the ION GNSS+ 2019.

